# Impact of Maternal High-Fat Diet on Offspring Cardiovascular–Kidney–Metabolic Health: Spotlight on Oxidative Stress

**DOI:** 10.3390/antiox14091136

**Published:** 2025-09-19

**Authors:** Chien-Ning Hsu, Chih-Kuang Chen, Chih-Yao Hou, Yu-Wei Chen, Guo-Ping Chang-Chien, Shu-Fen Lin, You-Lin Tain

**Affiliations:** 1 Department of Pharmacy, Kaohsiung Chang Gung Memorial Hospital, Kaohsiung 833, Taiwan; cnhsu@cgmh.org.tw; 2 Department of Pharmacy, Kaohsiung Municipal Ta-Tung Hospital, Kaohsiung 801, Taiwan; 3 School of Pharmacy, Kaohsiung Medical University, Kaohsiung 807, Taiwan; 4 Polymeric Biomaterials Laboratory, Department of Materials and Optoelectronic Science, National Sun Yat-sen University, Kaohsiung 804, Taiwan; chihkuan@mail.nsysu.edu.tw; 5 Department of Seafood Science, National Kaohsiung University of Science and Technology, Kaohsiung 811, Taiwan; chihyaohou@nkust.edu.tw; 6 Department of Food Science and Biotechnology, National Chung Hsing University, Taichung 402, Taiwan; d112043001@mail.nchu.edu.tw; 7 Department of Pediatrics, Kaohsiung Chang Gung Memorial Hospital, Kaohsiung 833, Taiwan; 8 Center for Environmental Toxin and Emerging-Contaminant Research, Cheng Shiu University, Kaohsiung 833, Taiwan; guoping@csu.edu.tw (G.-P.C.-C.); linsufan2003@csu.edu.tw (S.-F.L.); 9 Super Micro Mass Research and Technology Center, Cheng Shiu University, Kaohsiung 833, Taiwan; 10 Institute of Environmental Toxin and Emerging-Contaminant, Cheng Shiu University, Kaohsiung 833, Taiwan; 11 Department of Pediatrics, Kaohsiung Municipal Ta-Tung Hospital, Kaohsiung 801, Taiwan; 12 College of Medicine, Chang Gung University, Taoyuan 333, Taiwan

**Keywords:** oxidative stress, antioxidants, cardiovascular disease, hypertension, chronic kidney disease, obesity, diabetes, developmental origins of health and disease (DOHaD), metabolic syndrome, high-fat diet

## Abstract

Cardiovascular–kidney–metabolic syndrome (CKMS) encompasses interconnected cardiovascular, renal, and metabolic disorders, including obesity, hypertension, and type 2 diabetes. Oxidative stress is increasingly recognized as a central driver of this multi-organ dysfunction. Among maternal influences, exposure to a high-fat diet (HFD) during pregnancy and lactation consistently predisposes offspring to CKMS-related phenotypes in animal models. While oxidative stress is implicated as a key mediator, its precise role in developmental programming remains unclear, and comparing the differences in its role between overt CKMS and CKM programming is critical. Critical gaps include whether oxidative stress acts uniformly or in an organ- and time-specific manner, which signals initiate long-term redox alterations, and whether these effects are reversible. Furthermore, its interactions with other programming pathways—such as renin–angiotensin system activation, epigenetic dysregulation, gut microbiota imbalance, and altered nutrient sensing—remain insufficiently explored. This review uniquely highlights maternal HFD-induced oxidative stress as a mechanistic axis of CKMS programming and delineates unresolved questions that limit translation. By integrating evidence across organ systems and proposing priorities for multi-organ profiling, refined models, and longitudinal human studies, we outline a forward-looking agenda for the field. Ultimately, clarifying how maternal HFD and oxidative stress shape offspring CKMS risk is essential to inform targeted antioxidant strategies to reduce the intergenerational transmission of CKMS risk.

## 1. Introduction

Cardiovascular–kidney–metabolic syndrome (CKMS) refers to the interconnected pathophysiology linking cardiovascular disease (CVD), chronic kidney disease (CKD), and metabolic disorders such as obesity, type 2 diabetes, and insulin resistance [1]. It is estimated that nearly 90% of adults in the United States are affected by CKMS [2]. This syndrome is driven by shared mechanisms—including oxidative stress, systemic inflammation, endothelial dysfunction, and neurohormonal activation—that synergistically accelerate disease progression across organ systems [3]. Among these, oxidative stress serves as a key mechanistic link [4,5].

Understanding CKMS is clinically crucial, as early detection enables the avoidance of risk factors and provides an opportunity for preventive intervention [3]. A growing body of evidence suggests that adverse environmental exposures during pregnancy and early infancy increase susceptibility to CKD, CVD, obesity, and metabolic syndrome in later life—all components of CKMS [6,7,8,9]. According to the Developmental Origins of Health and Disease (DOHaD) theory, the fetus adapts to intrauterine environmental cues, which may predispose individuals to chronic diseases in adulthood [10]. However, these maladaptive programming effects can potentially be reversed by shifting therapeutic strategies from adulthood to the perinatal period—a concept known as reprogramming [11]. Given that CKMS may originate early in life, this DOHaD-based preventive approach represents a promising shift from treatment to early-life intervention.

Modern dietary patterns are strongly linked to the rising prevalence of CKMS-related conditions, including obesity, metabolic syndrome, CVD, and CKD [12,13]. Maternal nutrition plays a critical role not only in shaping fetal development but also in influencing long-term health trajectories [14,15]. In particular, maternal dietary imbalances have been shown to promote the developmental programming of oxidative stress pathways [16,17], which can persist postnatally and predispose offspring to CKMS [18]. These findings highlight the potential of maternal dietary modulation as a proactive strategy not only to reduce offspring risk but also to disrupt the intergenerational transmission of oxidative stress-related disease.

Animal models employing high-fat diets (HFDs) typically incorporate a variety of fat sources to simulate the Western dietary pattern, which contains approximately 70% more saturated fat than recommended by U.S. dietary guidelines [19]. These models are widely used to induce obesity, CVD, type 2 diabetes, and kidney dysfunction—hallmarks of CKMS [20,21,22,23,24]. Notably, HFDs are potent inducers of oxidative stress, exerting both direct effects on the pregnant mother and indirect programming effects on the developing fetus [16,17]. Excessive saturated fat intake during gestation disrupts maternal-fetal redox homeostasis, leading to elevated reactive oxygen species (ROS) levels that interfere with fetal organ development and long-term CKMS programming. A growing body of preclinical and human evidence links maternal HFD intake to heightened oxidative stress in the fetus and an increased predisposition to CKMS in the offspring [25,26,27]. Conversely, substituting saturated fatty acids (SFAs) with polyunsaturated fatty acids (PUFAs) has been associated with improved lipid profiles and reduced cardiovascular mortality [28].

Together, these findings highlight oxidative stress as a central mechanistic nexus through which maternal high-fat intake contributes to adverse offspring CKMS outcomes. The objective of this review is to critically evaluate how maternal HFD exposure influences offspring cardiovascular, kidney, and metabolic health, emphasizing oxidative stress as a central mechanistic driver and highlighting gaps in translating findings from animal models to humans.

## 2. Materials and Methods

This narrative review synthesizes existing evidence from both animal and human studies to assess the effects of maternal HFD on offspring CKMS, focusing specifically on oxidative stress as a key pathogenic mechanism. A comprehensive literature search was conducted using PubMed, Web of Science, and Scopus up to June 2025, using keywords related to maternal HFD, oxidative stress, DOHaD, and CKMS. Given the interdisciplinary nature and heterogeneity of available evidence, a narrative review format was chosen over a systematic or scoping approach to allow for a more integrative exploration of emerging concepts spanning developmental biology, redox medicine, clinical sciences, and maternal-fetal nutrition.

Search terms included combinations of the following keywords: “high-fat diet”, “saturated fat”, “dietary fat”, “Western diet”, “maternal diet”, “perinatal nutrition”, “polyunsaturated fatty acids”, “omega-3 fatty acids”, “metabolic syndrome”, “obesity”, “chronic kidney disease”, “cardiovascular disease”, “hypertension”, “hyperlipidemia”, “dyslipidemia”, “insulin resistance”, “hyperglycemia”, “diabetes”, “liver steatosis”, “atherosclerosis”, “heart failure”, “developmental programming”, “DOHaD”, “offspring”, “progeny”, “mother”, “oxidative stress”, “reactive oxygen species”, “reactive nitrogen species”, “nitric oxide”, “antioxidant”, “reprogramming”, “pregnancy”, and “lactation”.

Studies were included if they examined maternal dietary fat intake during pregnancy or lactation and its effects on oxidative stress and CKMS-related outcomes in offspring. Both experimental models and human observational studies were reviewed to evaluate the role of ROS, redox imbalance, and interventions such as PUFA supplementation. Due to variability in study designs and endpoints, findings were synthesized narratively, with a focus on elucidating underlying mechanisms and informing early-life nutritional strategies for CKMS prevention.

## 3. High-Fat Diets, Oxidative Stress, and CKMS

### 3.1. Dietary Fats

Dietary fats are primarily triglycerides, composed of glycerol and fatty acids, and are commonly referred to as ‘fats’ when solid and ‘oils’ when liquid at room temperature [29]. Triglycerides are a subset of lipids, which represent a broader class of fat-related biomolecules. Fatty acids vary by chain length and degree of saturation: SFAs have only single bonds, whereas unsaturated fatty acids contain one (monounsaturated) or more (polyunsaturated) double bonds, which may exist in *cis* or *trans* configurations. Naturally occurring fatty acids are typically in the *cis* form, while trans fats, especially industrially produced ones, are linked to adverse health outcomes [24,25].

Essential PUFAs, including linoleic acid (C18:2 n-6, LA) and alpha-linolenic acid (C18:3 n-3, ALA), are required for normal physiological functions. Saturated and *trans* fats are associated with increased cardiovascular risk, whereas monounsaturated and polyunsaturated fats are generally protective [30]. Once ingested, fats are absorbed in the intestine and reassembled into triglycerides and cholesteryl esters. These hydrophobic molecules require transport by lipoproteins [31]. Chylomicrons, produced in the intestine, deliver triglycerides to peripheral tissues, forming chylomicron remnants. In the liver, very low-density lipoproteins (VLDL) are synthesized and converted to low-density lipoproteins (LDL), which transport cholesterol [32]. Oxidized LDL can contribute to vascular pathology. In contrast, high-density lipoproteins (HDLs) facilitate reverse cholesterol transport, removing cholesterol from tissues and offering cardiovascular protection.

### 3.2. Oxidative Stress

Oxidative stress refers to an imbalance between the production of ROS and NO, and the capacity of the body’s antioxidant systems to neutralize them [33]. ROS include both free radicals (e.g., superoxide anion and hydroxyl radical) and non-radical species like hydrogen peroxide, primarily generated by enzymes such as NADPH oxidases, xanthine oxidase, and the mitochondrial respiratory chain [34]. Reactive nitrogen species (RNS), such as peroxynitrite and nitrogen dioxide, are formed when NO reacts with superoxide, especially under conditions of excessive oxidative burden [35,36]. While NO plays essential physiological roles as a vasodilator and signaling molecule [37], its bioavailability is diminished when nitric oxide synthase (NOS) becomes uncoupled—often due to elevated levels of asymmetric dimethylarginine (ADMA)—leading to further ROS and RNS production [38]. The body counters oxidative stress through enzymatic antioxidants (e.g., SOD, catalase) and non-enzymatic defenses (e.g., glutathione, vitamins) [39]. Maintaining a balanced ROS and NO environment is essential not only for cardiovascular and kidney function but also for preserving metabolic homeostasis and BP regulation [40,41,42].

### 3.3. Interplay Between High-Fat Diets, Oxidative Stress, and Pathogenesis of CKMS

While dietary lipids are crucial for normal physiological function, excessive intake—particularly of saturated and *trans* fats—has become a significant environmental factor in the global rise in various disorders, including obesity, hypertension, type 2 diabetes, dyslipidemia, CKD, and CVD [20,21,22,23,24]. HFDs exert widespread effects that disrupt metabolic, cardiovascular, and renal homeostasis, thereby driving the onset and progression of CKMS.

The pathogenesis of CKMS is mediated by interconnected mechanisms—including oxidative stress, chronic systemic inflammation, endothelial dysfunction, and neurohormonal dysregulation—that collectively accelerate organ injury and metabolic derangement [1,3]. Among these, oxidative stress plays a central mechanistic role, acting both as a primary driver and as an amplifier of inter-organ pathology.

The following section provides an integrative overview of how HFDs influence various organ systems, with a particular emphasis on oxidative stress as a unifying factor and mediator of crosstalk among metabolic, cardiovascular, and renal pathways in the development of CKMS.

#### 3.3.1. Adipose Tissue

A HFD promotes obesity and metabolic syndrome primarily by disrupting adipose tissue function. Excess fat intake leads to adipocyte hypertrophy, triggering inflammation through increased secretion of pro-inflammatory cytokines and macrophage infiltration [43]. This inflammatory state impairs insulin signaling and alters adipokine secretion—decreasing protective adiponectin and increasing leptin, often leading to leptin resistance. Insulin-resistant adipose tissue increases lipolysis, releasing FFAs that accumulate in the liver, muscle, and pancreas, contributing to systemic insulin resistance and lipotoxicity [44].

Lipotoxicity is the detrimental effect of excess lipid accumulation in non-adipose tissues, including the liver, muscle, heart, pancreas, and kidney. When adipose storage capacity is exceeded, free fatty acids and lipid intermediates build up, disrupting cellular function. This induces mitochondrial and ER stress, leading to increased ROS production and activation of pro-inflammatory pathways, which further damage proteins, lipids, and DNA. Consequently, lipotoxicity and oxidative stress create a self-reinforcing cycle that promotes organ dysfunction and contributes to the development of CKMS [43,44,45].

Additionally, high-fat intake suppresses thermogenesis and energy expenditure by inhibiting brown and beige fat activity, reinforcing energy imbalance and fat accumulation. These changes collectively drive the development of central obesity, insulin resistance, dyslipidemia, and hypertension—hallmarks of CKMS.

In addition to promoting inflammation, HFD increases ROS production in adipose tissue through mitochondrial overload, NADPH oxidase activation, and endoplasmic reticulum stress [45]. The resulting oxidative stress, due to an imbalance between ROS and antioxidant defenses, further impairs insulin signaling (e.g., via IRS-1/PI3K/AKT) and activates NF-κB-mediated inflammatory pathways [46,47]. This redox imbalance also contributes to adipocyte apoptosis, fibrosis, and further immune cell infiltration, worsening adipose tissue dysfunction. Lipid peroxidation products generated under oxidative stress can damage peripheral organs, amplifying the systemic effects of CKMS [48,49]. The transcription factor nuclear factor E2-related factor 2 (Nrf2) regulates antioxidant defenses, modulates adipocyte differentiation, and controls liver energy metabolism while suppressing lipid synthesis. HFDs reduce Nrf2 mRNA and downstream targets, highlighting the influence of dietary fat composition on oxidative stress susceptibility [50].

In summary, HFD-induced adipose dysfunction arises from a dual hit of inflammation and oxidative stress. Together, these processes disrupt insulin signaling, alter energy balance, and propagate systemic metabolic injury, thereby establishing a mechanistic link between excess fat intake and the development of CKMS.

#### 3.3.2. Liver, Pancreas, and Skeletal Muscle

A HFD induces dyslipidemia and fatty liver by overwhelming the body’s lipid-handling capacity [51,52]. Excess dietary fat increases FFA influx to the liver from both diet and adipose tissue lipolysis, leading to triglyceride accumulation and hepatic steatosis [53]. Insulin-driven de novo lipogenesis, impaired β-oxidation, and oxidative stress further disrupt lipid metabolism. Increased hepatic VLDL production raises plasma triglycerides and lowers HDL, characteristic of dyslipidemia. Insulin resistance reduces lipoprotein lipase activity, impairing lipid clearance. Ectopic fat deposition in liver, muscle, and pancreas contributes to insulin resistance and β-cell dysfunction, key features of CKMS [54].

Simultaneously, lipid accumulation in skeletal muscle, where they generate lipid intermediates such as diacylglycerol and ceramides that impair insulin signaling via IRS-1/PI3K/AKT inhibition [55,56]. The pancreas responds by increasing insulin secretion, but chronic lipotoxicity and glucotoxicity ultimately cause β-cell dysfunction and apoptosis, impairing glucose regulation [57]. Meanwhile, reduced HDL and elevated VLDL levels promote endothelial dysfunction, contributing to hypertension and vascular injury [58]. Together, these organ-specific disturbances converge to drive the progression of central obesity, insulin resistance, type 2 diabetes, and cardiovascular–kidney–metabolic complications characteristic of CKMS [22].

In summary, HFD-induced dyslipidemia and ectopic lipid deposition disrupt hepatic, muscular, pancreatic, and vascular function. This multi-organ lipid overload establishes a pathogenic cascade that links excess fat intake to systemic insulin resistance, β-cell failure, and the cardiometabolic complications that define CKMS.

#### 3.3.3. Kidney

A HFD contributes to kidney disease through multiple interrelated mechanisms [31]. Excess fat intake induces obesity and insulin resistance, which activate the renin–angiotensin system (RAS), increase sympathetic activity, and elevate blood pressure (BP)—factors that promote glomerular hypertension and hyperfiltration, initiating renal injury [59,60]. HFD also leads to ectopic lipid accumulation in the kidney (renal lipotoxicity), causing tubular injury, podocyte dysfunction, and glomerulosclerosis [61]. Concurrently, HFD-induced oxidative stress and inflammation activate pro-fibrotic pathways, such as TGF-β signaling, accelerating renal fibrosis and functional decline [62]. Gut-derived endotoxins (e.g., LPS), elevated in HFD-induced dysbiosis, further aggravate systemic and renal inflammation via TLR4/NF-κB signaling [63,64]. Mitochondrial dysfunction amplifies oxidative stress by overproducing ROS, reducing ATP generation, and activating inflammatory and apoptotic pathways, thereby promoting cellular and organ injury. In renal cells, impaired mitochondrial function and fatty acid oxidation exacerbate oxidative damage and energy deficits, driving CKD progression and reinforcing the cardiovascular–kidney–metabolic disturbances characteristic of CKMS [65].

Collectively, HFD promotes renal injury via hemodynamic stress, lipid toxicity, oxidative stress, and inflammation. These mechanisms converge to accelerate CKD progression and integrate kidney dysfunction into the broader cardiometabolic network of CKMS.

#### 3.3.4. Cardiovascular System

A HFD rich in saturated fatty acids is a risk factor for atherosclerosis, which commonly precipitates as ischemic events, transient ischemic attacks, and myocardial infarction [66]. A HFD promotes cardiovascular injury by driving oxidative stress, inflammation, and lipid imbalance. Excess dietary fat increases circulating FAAs and promotes lipid accumulation in vascular and cardiac tissues, leading to mitochondrial overload and oxidative stress. HDF-induced oxidative stress not only overproduce ROS but also reduces nitric oxide (NO) bioavailability, impairs endothelial function, and triggers endothelial cell apoptosis—early steps in vascular dysfunction [67]. ROS also activate redox-sensitive signaling pathways such as NF-κB and MAPK, promoting vascular inflammation, monocyte recruitment, and atherosclerotic plaque formation [68]. In parallel, oxidative stress enhances oxidized LDL formation, which exacerbates endothelial injury and foam cell development.

HFD-induced ROS generation in the heart contributes to cardiomyocyte hypertrophy, mitochondrial dysfunction, and myocardial fibrosis, impairing cardiac contractility and diastolic function [69]. Furthermore, oxidative stress activates the RAS and sympathetic nervous system, promoting vasoconstriction and hypertension [70]. These oxidative and inflammatory cascades synergize to drive hypertension, vascular stiffness, and cardiac remodeling—central features of the cardiovascular axis of CKMS [22].

In summary, HFD-driven oxidative stress and inflammation orchestrate vascular dysfunction, atherosclerosis, and cardiac remodeling, positioning oxidative stress as a unifying mechanism in the cardiovascular axis of CKMS.

#### 3.3.5. Inter-Organ Crosstalk in CKMS Pathogenesis

A HFD disrupts inter-organ communication by triggering oxidative stress, systemic inflammation, and widespread metabolic dysregulation. In adipose tissue, HFD induces adipocyte hypertrophy and inflammation, leading to increased secretion of pro-inflammatory cytokines and reduced adiponectin. These adipokine alterations impair insulin sensitivity in the liver, promote lipogenesis, and contribute to hepatic steatosis. Elevated circulating FFAs and inflammatory mediators also affect skeletal muscle, reducing glucose uptake and exacerbating insulin resistance. In the pancreas, chronic lipid overload and oxidative stress impair β-cell function and insulin secretion. Concurrently, lipid accumulation induces endoplasmic reticulum (ER) stress, activating the unfolded protein response and amplifying oxidative stress, apoptosis, and endothelial dysfunction [71]. This feed-forward loop between lipid signaling and ER stress under HFD exposure contributes to organ injury [72], metabolic disturbances, and the developmental programming of CKMS.

Dysfunction of the liver–muscle–adipose axis amplifies systemic insulin resistance and dyslipidemia, both of which contribute to kidney injury through glomerular hyperfiltration, lipid deposition, and oxidative damage. Concurrently, HFD-induced gut dysbiosis increases intestinal permeability and promotes translocation of LPS into circulation. LPS activates TLR4/NF-κB signaling pathways across multiple organs—including the liver, vasculature, kidney, and brain—fueling chronic low-grade inflammation. In the central nervous system, hypothalamic inflammation and leptin resistance impair appetite regulation and autonomic balance, promoting sympathetic overactivity, hypertension, and renal vasoconstriction.

**Figure 1:**
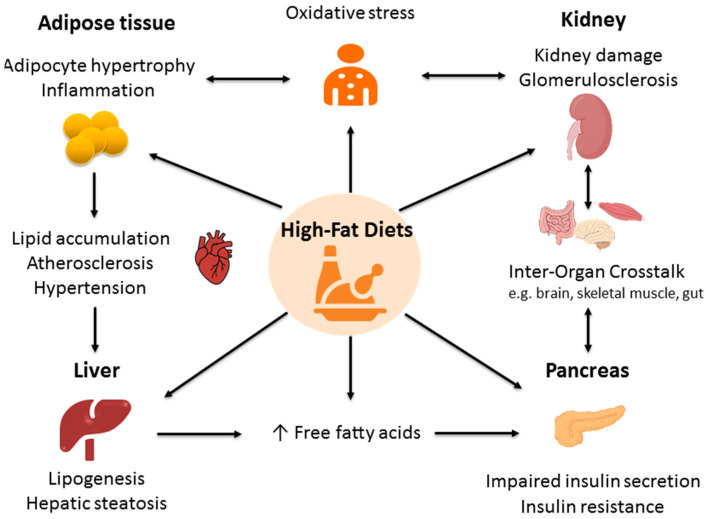
Organ-specific pathologies and inter-organ crosstalk associated with exposure to a high-fat diet. ↑ = increased.

Collectively, these maladaptive inter-organ feedback loops—driven by excess lipids, ROS, cytokines, and neurohormonal signals—establish a self-reinforcing cycle of progressive multisystem injury [73]. Oxidative stress in one organ can propagate dysfunction in others through circulating FFAs, inflammatory mediators, and redox-sensitive signaling, illustrating how organ-specific damage is amplified across the metabolic, renal, and cardiovascular systems. A HFD thus acts as a systemic metabolic disruptor, with both organ-specific injury and disrupted inter-organ crosstalk synergistically driving CKMS pathogenesis. Figure 1 is a schematic illustration adapted from published literature [31,45,49,59,64], highlighting redox-mediated inter-organ feedback loops and integrating mechanistic insights across adipose tissue, liver, muscle, pancreas, kidney, and cardiovascular systems. This figure provides a comprehensive visual summary of how oxidative stress links organ-specific pathologies and inter-organ interactions, complementing the detailed mechanistic explanations presented in the text.

## 4. Distinct Roles of Oxidative Stress in CKMS and CKM Programming

Oxidative stress plays distinct roles across the spectrum of CKMS conditions, reflecting its impact at different life stages. In overt CKMS, oxidative stress acts as a direct pathogenic driver, promoting organ dysfunction and disease progression. In contrast, during CKM programming, which arises from early-life exposures, oxidative stress exerts an indirect, developmental influence—modulating organogenesis, metabolic signaling, and epigenetic regulation during critical developmental windows [74,75,76,77]. These early redox imbalances shape long-term physiological trajectories, increasing susceptibility to CKMS later in life. Recognizing these stage-specific roles is key to designing interventions aimed at both preventing early-life programming and managing established disease.

### 4.1. The Role of Oxidative Stress in CKMS

CKMS is classified into four progressive stages (1–4), each reflecting increasing severity across metabolic, renal, and cardiovascular systems [1]. Stage 1 involves individuals with excessive or abnormal fat distribution, where HFDs promote ectopic lipid accumulation in adipose, liver, and muscle [54], leading to elevated ROS, adipose inflammation, and reduced NO bioavailability—early events that disrupt vascular and metabolic homeostasis. In Stage 2, defined by the emergence of metabolic syndrome features such as dyslipidemia, hypertension, and hepatic steatosis, oxidative stress perpetuates low-grade inflammation through redox-sensitive pathways like NF-κB [5,45], while lipid peroxidation and ROS-induced damage impair liver, kidney, and pancreatic β-cell function. By Stage 3, subclinical cardiovascular and renal injury become evident: sustained oxidative stress contributes to cardiac hypertrophy [78], vascular stiffening [79], and CKD [80]. In Stage 4, characterized by overt CKD, CVD, and type 2 diabetes, oxidative stress emerges as a central driver of irreversible organ damage, perpetuating a vicious cycle by amplifying ischemic injury, destabilizing atherosclerotic plaques, and accelerating β-cell dysfunction and failure [81,82]. In summary, oxidative stress operates across all four CKMS stages—as both an initiator and amplifier of multisystem dysfunction—making it a central therapeutic target to interrupt disease progression.

### 4.2. The Role of Oxidative Stress in CKM Programming

During pregnancy, the physiological generation of ROS is essential for key developmental processes, including oocyte maturation, embryo implantation, placental formation, and fetal organogenesis [83,84,85]. Fetal oxygen requirements vary across trimesters, starting low in the first trimester and increasing in later stages due to fetal growth and the establishment of the fetal–placental circulation [86]. However, pregnancies complicated by maternal illness or environmental insults are often accompanied by oxidative stress, which can disrupt normal development and induce long-term alterations in fetal physiology—a phenomenon referred to as developmental programming—thereby increasing the risk of chronic disease in adulthood [87].

Given the multisystem nature of CKMS, numerous animal models have been developed to replicate different facets of CKMS in adult offspring [88]. Despite variations in the maternal insults used to induce disease, oxidative stress consistently emerges as a key early-life risk factor linking these exposures to offspring CKMS outcomes.

Mechanistically, oxidative stress in CKM programming is characterized by a constellation of molecular alterations, including upregulation of ROS-generating enzymes [89], excessive ROS production [90], reduced antioxidant capacity [91], impairment of the ADMA–NO pathway [92], and cumulative oxidative damage to cellular components [93]. Biomarkers of oxidative damage—such as malondialdehyde (MDA) [94], thiobarbituric acid reactive substances (TBARS) [95], F2-isoprostanes [96], 4-hydroxynonenal (4-HNE) [97], and 8-hydroxydeoxyguanosine (8-OHdG) [98]—have been widely used to validate these observations in animal models of CKMS programming.

In addition to redox imbalance, oxidative stress orchestrates CKM programming through its interplay with fundamental pathways such as aberrant RAS signaling [99,100], epigenetic dysregulation [101], disturbances in gut microbiota composition [102], and dysregulated nutrient-sensing signals [16].

RAS components are highly expressed during fetal development and play critical roles in kidney and cardiovascular organogenesis [103,104]. Oxidative stress activates the classical RAS axis (ACE–Ang II–AT1R), creating a self-amplifying loop: Ang II stimulates NADPH oxidase to generate ROS, which in turn enhances RAS activity. This maladaptive cycle promotes inflammation, vasoconstriction, and fibrosis—hallmarks of CKMS. Meanwhile, the protective ACE2–Ang-(1–7)–MAS axis is suppressed by oxidative insults. Notably, early postnatal intervention targeting this axis—prior to full renal maturation—can attenuate programmed RAS overactivity without impairing normal development [100].

Oxidative stress also interfaces with epigenetic regulation. Alterations in DNA methylation, histone modifications, and microRNA expression have been implicated in CKMS-related phenotypes such as CVD, type 2 diabetes, and CKD [105,106,107]. Organs with high epigenetic plasticity, including the kidney, liver, and adipose tissue, are particularly vulnerable to redox imbalance during early life [108]. For example, maternal HFD exposure induces ROS-mediated hypomethylation of ACE and AT1R promoters, enhancing their expression [109,110], while ROS can impair DNA methyltransferase activity, disrupting adipogenic differentiation and methylation profiles in fat progenitor cells [111], thereby increasing obesity risk. Importantly, oxidative stress modulates the activity of peroxisome proliferator-activated receptors (PPARs) [112], which regulate lipid metabolism, inflammation, and redox balance. Maternal nutritional insults can program renal structure and function via the PPAR–oxidative stress axis [112], with PPARs controlling kidney development genes [77], antioxidant pathways [113], and sodium transporters [114]. ROS further amplifies these effects by altering PPAR activity and epigenetic regulators, driving nephron deficits and programmed hypertension [77]. Moreover, ROS-induced epigenetic modifications of PPARα and PPARγ [115] may dysregulate metabolic and inflammatory signaling in the kidney, liver, and adipose tissue, amplifying CKMS susceptibility.

Another pivotal pathway is gut microbiota dysbiosis. Maternal factors shape the initial colonization and composition of the offspring’s gut microbiome, with long-lasting impacts on metabolic and immune function [116,117]. Oxidative stress alters microbial ecology and disrupts gut barrier integrity. Maternal insults lead to microbial dysbiosis in offspring, reducing beneficial metabolites such as short-chain fatty acids while increasing harmful compounds like trimethylamine N-oxide (TMAO) and uremic toxins [118,119,120,121]. These changes reinforce systemic oxidative stress, creating a bidirectional loop that disrupts redox homeostasis and immune signaling—further entrenching CKMS traits. Oxidative injury to the intestinal epithelium also promotes endotoxemia and systemic inflammation, further exacerbating disease risk [122].

Dietary fats can modulate nutrient-sensing pathways that regulate lipid detection, satiety, food intake, and weight gain [123,124]. Key nutrient-sensing signals include AMP-activated protein kinase (AMPK) [125], sirtuin-1 (SIRT1) [126], PPARs [127], and PPARγ coactivator-1α (PGC-1α) [128]. In particular, lipid-sensing nuclear receptors such as PPARs and PGC-1α play central roles in lipid metabolism [129]. Since maternal diet influences fetal metabolism and development through these pathways, disruptions during compromised pregnancies can impair both maternal and fetal nutrient-sensing mechanisms—contributing to the developmental origins of adult diseases [130,131]. Dysregulation of nutrient-sensing pathways—particularly involving AMPK, PPARs, and PGC-1α—has been implicated in the pathogenesis of various CKMS conditions [132,133,134,135]. Moreover, emerging evidence highlights the interplay between oxidative stress and nutrient-sensing pathways in CKMS programming [16]. Therefore, targeting these nutrient-sensing signals may represent a promising reprogramming strategy to prevent the onset of CKMS.

Understanding oxidative stress as a central integrator of multiple pathogenic pathways in CKM programming underscores its potential as a therapeutic target. However, the role of oxidative stress in programming CKMS differs from its function in established, overt disease. In programming, it acts as a developmental trigger, while in overt CKMS, it contributes to disease progression and end-organ damage. Key distinctions—particularly in how the oxidative stress contributes to CKM programming and overt CKMS—are illustrated in Table 1.

## 5. CKMS of Developmental Origins: The Impact of Maternal High-Fat Diet

### 5.1. Human Evidence

In humans, maternal diet, particularly high-fat patterns, can influence children’s dietary habits and may contribute to adverse health outcomes [136,137]. However, no study has yet directly addressed the impact of maternal HFDs on child outcomes such as obesity, metabolic disorders, or CKMS.

Certain diets benefit cardiovascular–kidney–metabolic health. Calorie restriction, with or without time-restricted eating, similarly reduces body weight in obesity [138]. The ketogenic diet improves glucose, lipid control, and weight in T2DM [139] but is unsuitable during pregnancy. In contrast, plant-based and Mediterranean diets lower T2DM and obesity risk and are considered optimal in pregnancy [140,141]. However, the impact of maternal fat-patterned diets on offspring outcomes remains unclear. Given that most epidemiological studies involve diverse populations and mixed dietary fat sources—potentially diluting meaningful associations—animal models are essential for uncovering the mechanistic pathways of maternal HFD-induced developmental programming.

### 5.2. Animal Models of CKMS of Developmental Origins

Although HFDs are widely used in animal studies to induce obesity and related disorders [26], the term lacks a standardized definition [142,143]. Fat content in these diets can vary widely—from 20% to 60% of total energy—and the fat sources range from animal fats (e.g., lard, butter) to plant oils (e.g., corn, coconut oil). Furthermore, the health effects may differ depending on whether the fats are predominantly saturated or unsaturated [144]. Due to this variability, maternal HFD-induced phenotypes can differ substantially across animal studies.

Multiple species have been employed to examine the impact of maternal HFDs on offspring outcomes, including small animals such as rats, mice [26], and rabbits [145], as well as large animals like pigs [146] and non-human primates [147]. As reviewed by our group and others [17,25,26,27,31,148,149], maternal HFD exposure has been linked to altered offspring feeding behavior, changes in body composition, and increased risks of type 2 diabetes, obesity, insulin resistance, hepatic steatosis, dyslipidemia, hypertension, and kidney disease—all hallmarks of CKMS.

Although many animal studies have investigated maternal HFDs and offspring health [20,21,60,61], only a limited number have focused specifically on oxidative stress. This review centers on those studies that explicitly examined oxidative stress as a mechanism contributing to CKMS outcomes. Table 2 summarizes preclinical studies recording offspring’s CKMS outcomes related to oxidative stress in which maternal HFDs were applied during gestation and lactation [95,150,151,152,153,154,155,156,157,158,159,160,161,162,163,164,165].

Table 2 summarizes maternal HFDs with fat content ranging from 23% to 58% of energy, consistent with prior studies [142,143]. However, commonly used rodent HFDs often differ from typical Western diets, which generally contain lower fat and protein levels [149]. Beyond purified HFDs, human-relevant dietary models such as Western, Western-style, or cafeteria diets have also been employed in studies of CKMS [166,167]. Nevertheless, limited evidence exists on whether these diets elicit oxidative stress-related effects comparable to those induced by conventional HFDs in CKMS programming.

Another concern is whether a dose-dependent relationship exists between maternal fat intake and oxidative stress or CKMS outcomes. A systematic review and meta-analysis of 77 rodent studies demonstrated that maternal HFD exposure during pregnancy and/or lactation significantly elevates oxidative stress markers in offspring. While excessive oxidative stress can lead to organ injury and adverse outcomes [17], variability in HFD sources, concentrations, and exposure periods across studies introduces substantial heterogeneity, making it impossible to perform a meta-analysis to definitively determine dose-dependent effects. Our review highlights the need for further research to clarify whether higher maternal fat intake correlates with greater oxidative damage and more severe CKMS outcomes in offspring.

While maternal obesity is frequently modeled in rodents using HFDs, it is important to note that the developmental programming effects of maternal obesity and maternal high-fat exposure are not identical [149]. In most studies aiming to induce maternal obesity, HFDs were initiated 4–9 weeks prior to mating [160,161], underscoring the need to distinguish between pre-pregnancy obesity and diet-induced metabolic programming.

Rodent studies commonly use diets enriched in SFAs and, to a lesser extent, monounsaturated fatty acids (MUFAs). Key SFAs include palmitic acid and lauric acid (C12:0), typically derived from lard, coconut oil, and palm oil, whereas oleic acid (C18:1n-9) is a representative MUFA. The type and proportion of fatty acids are crucial, as SFAs strongly induce oxidative stress, inflammation, and insulin resistance in offspring, promoting CKMS-related phenotypes. MUFAs, in contrast, may exert relatively protective effects by modulating redox balance and lipid metabolism. Highlighting the specific fatty acid composition helps clarify mechanistic links between maternal diet, oxidative stress, and organ-specific programming of metabolic, cardiovascular, and renal dysfunction in offspring.

As shown in Table 2, the effects of maternal HFDs on rat offspring were evaluated from 9 to 32 weeks of age—corresponding to human adolescence through early adulthood [168]. These CKMS-related phenotypes include kidney disease [98,150,155,158,159,160,164], obesity [151,153,159,160,161], dyslipidemia [151,152,158,160,162,163], hyperglycemia [159,160], insulin resistance [151,152,156,159,160], hepatic steatosis [153,156,162,163], endothelial dysfunction [161], and hypertension [151,152,154,155,157,161,164,165]. Notably, the CKMS phenotypes induced by maternal HFDs vary considerably depending on offspring age, rodent species, and the type and proportion of fatty acids in the maternal diet.

Emerging evidence suggests that the development of complex conditions such as CKMS often results from a cumulative sequence of insults, commonly referred to as the ‘multiple-hit hypothesis’ [169]. Drawing from the DOHaD framework, an individual’s long-term health trajectory can be shaped by a cascade of stressors occurring during sensitive developmental windows and continuing throughout life [170]. The initial, or ‘first hit,’ typically stems from adverse maternal exposures that predispose the fetus to future disease susceptibility. Subsequent ‘second hits,’ encountered after birth, may act as triggers that unmask or intensify these latent vulnerabilities.

Experimental models have utilized maternal HFDs as a primary prenatal insult, followed by additional postnatal challenges to provoke disease phenotypes in adulthood. Examples include sequential exposure to HFDs during both maternal and post-weaning periods [164,171], or the combination of maternal high-fat intake with high-sucrose or high-fructose consumption [64]. These layered exposures may activate converging pathophysiological mechanisms, leading to synergistic or amplified effects that culminate in overt disease.

Collectively, studies employing diverse maternal HFD compositions—particularly those rich in saturated fats—support the notion that such dietary environments adversely program offspring toward increased risk of cardiovascular, kidney, and metabolic disorders.

### 5.3. Oxidative Stress as a Central Link Between Maternal HFD to Offspring CKMS

A growing body of evidence highlights oxidative stress as a critical mediator in the developmental programming of CKMS following maternal HFD exposure. Maternal HFDs have been shown to induce both systemic and organ-specific oxidative stress in offspring, characterized by increased ROS generation [161,163], upregulation of ROS-producing enzymes [156], elevated lipid peroxidation [151,152,154,162], decreased NO bioavailability [161,164,165], and impaired antioxidant defenses, including reductions in SOD, catalase, and glutathione peroxidase (GPx) activities [151,152,153,162]. These redox imbalances adversely affect key organs implicated in CKMS pathogenesis, notably the kidneys [98,150,154,155,158,159], liver [153,154,162,163], vasculature [151,152,161], and pancreas [156].

In the kidneys, maternal HFD exposure disrupts nephrogenesis, leading to glomerular hypertrophy, podocyte injury, and tubulointerstitial fibrosis. These alterations are frequently associated with reduced expression of SIRT1, a pivotal regulator of redox balance and mitochondrial function [158]. Experimental interventions—such as GLP-1 receptor agonists [150], hydralazine [159], resveratrol [164], and 5-aminoimidazole-4-carboxamide riboside (AICAR), an AMPK activator [165]—have shown efficacy in ameliorating oxidative damage and restoring redox homeostasis in the offspring kidneys.

Sex-specific differences critically influence the oxidative stress response. Maternal and post-weaning HFDs program renal outcomes in a sex-dependent manner, with males more susceptible to hypertension and kidney injury through heightened oxidative stress, reduced NO bioavailability, and metabolic dysfunction, whereas females, despite greater transcriptomic sensitivity, appear relatively protected via estrogen-mediated resilience and adaptive pathways [98]. Similarly, maternal HFD induced insulin resistance in both sexes, but only males developed β-cell dysfunction with impaired insulin secretion and oxidative stress, while females were protected by higher estradiol and lower islet oxidative damage [156]. In contrast, a maternal lard-rich diet caused endothelial dysfunction in both sexes, yet only females exhibited sustained hypertension, likely driven by heightened sympathetic activity, altered HPA axis programming, and insulin resistance [154]. Collectively, these findings underscore unresolved questions about hormonal modulation of redox pathways and emphasize the need for sex-specific preventive strategies targeting oxidative stress early in life.

Postnatal dietary exposures act as a second hit, exacerbating oxidative stress and accelerating CKMS progression. Offspring exposed to both prenatal and postnatal HFDs show compounded impairments in renal and metabolic function, consistent with a “two-hit” model where prenatal redox priming sensitizes tissues to postnatal insults [98,160]. Additionally, maternal HFDs epigenetically repress antioxidant gene transcription and activate cellular senescence pathways in the liver and kidney, reinforcing the concept that oxidative dysregulation is heritably programmed [162,163,172].

Conversely, perinatal dietary interventions—particularly with PUFAs—have shown potential for reprogramming offspring outcomes. Supplementation with PUFAs during pregnancy and lactation has been reported to mitigate features of CKMS in adult offspring, including hypertension [173], cardiovascular dysfunction [174], and hepatic steatosis [175]. Conjugated linoleic acid (CLA), a derivative of dietary PUFAs such as LA, has been shown to protect against maternal high saturated fat diet-induced hypertension in rat models [173]. Despite existing recommendations for PUFA intake during pregnancy and breastfeeding [176], a meta-analysis of over 3600 children concluded that maternal omega-3 supplementation does not significantly reduce offspring obesity risk [177]. Whether perinatal intake of unsaturated fats can effectively counteract the programming effects of saturated fat—particularly via modulation of oxidative stress—remains an open question requiring further investigation.

Collectively, these findings position oxidative stress as a central and converging mechanism linking maternal HFDs to long-term offspring CKMS risk. Early-life interventions that enhance antioxidant defenses or attenuate ROS production may hold promise in interrupting the intergenerational cycle of CKMS.

## 6. Targeting Maternal HFD-Induced Oxidative Stress: Antioxidants in CKMS Prevention

Oxidative stress represents a key mechanistic link between maternal HFDs and the developmental programming of CKMS in offspring. Accordingly, early-life antioxidant interventions have emerged as a promising approach to mitigate or prevent CKMS outcomes.

Antioxidants, obtained from either dietary intake or synthetic sources, play a central role in counteracting oxidative damage [178]. The U.S. Institute of Medicine defines dietary antioxidants as compounds that (1) are regularly consumed through the human diet, (2) are present in appreciable amounts in common foods, and (3) exhibit measurable physiological effects in reducing oxidative injury [179]. Naturally occurring antioxidants—including vitamins C and E, polyphenols (e.g., resveratrol, quercetin), and amino acid derivatives such as L-citrulline and L-arginine—have shown protective effects against CKMS-related outcomes [18,180], though only polyphenols have been applied in maternal HFD models.

Resveratrol improves NO bioavailability, reduces oxidative stress markers, and reverses HFD-induced kidney dysfunction, obesity, hypertension, and metabolic disturbances in offspring [164,171,181,182], while quercetin protects against HFD-induced offspring hypertension [183]. Antioxidant-rich dietary patterns, such as the Mediterranean diet with high olive oil polyphenols, are associated with reduced risk of programmed kidney injury and hypertension in experimental models [184,185].

Melatonin, a potent endogenous antioxidant, protects against offspring hypertension and liver steatosis by neutralizing free radicals and restoring antioxidant systems [186,187]; though not a dietary antioxidant, it remains a promising prenatal intervention, with clinical use limited by safety data in pregnancy [188,189].

Synthetic antioxidants, including N-acetylcysteine (NAC) and MitoQ, replenish glutathione and target mitochondrial oxidative stress, respectively [190,191], and maternal NAC supplementation during lactation improves metabolic profiles, including body weight and hepatic steatosis, in adult offspring exposed to maternal HFDs [192]. Table 3 summarizes current evidence on perinatal antioxidant interventions as a reprogramming strategy to protect offspring from CKMS in HFD animal models.

In summary, antioxidant-based reprogramming strategies targeting maternal HFD-induced oxidative stress offer strong preclinical evidence for the prevention of CKMS. Nevertheless, further translational studies and clinical trials are essential to determine their efficacy, optimal timing, and safety in human populations.

## 7. Research Gaps and Future Directions

Maternal exposure to a HFD during pregnancy and lactation is consistently associated with an increased risk of CKMS phenotypes in offspring, including obesity, kidney disease, hypertension, CVD, and type 2 diabetes, primarily demonstrated in animal models. However, direct causal evidence in humans remains limited. Although oxidative stress is recognized as a key mediator of these outcomes, its precise functional role in developmental programming is not fully understood. Manipulating redox balance during critical developmental stages remains technically and biologically challenging, particularly given that susceptibility to oxidative stress varies among organs. As a result, maternal HFD exposure may induce distinct organ-specific programming effects.

We recognize that the effects of dietary fats on oxidative stress extend beyond total fat intake. Fat source, including the distinction between saturated and unsaturated fats or plant- versus animal-based fats, can significantly influence lipid peroxidation and ROS generation. Moreover, the broader dietary pattern in which fats are consumed—for example, Western versus Mediterranean diets—modulates oxidative stress through interactions with other nutrients and bioactive compounds. Cooking and processing techniques, such as frying, grilling, or high-temperature treatment, further alter fat composition and enhance the formation of oxidized lipids, amplifying oxidative damage. Together, these factors highlight that both the quality and context of fat intake are critical determinants of oxidative stress and its downstream consequences, and should be carefully considered in studies examining HFD-induced programming of CKMS risk.

Several important questions remain unanswered: Does oxidative stress act uniformly across organs, or are its effects organ- and time-specific? Which specific free radical signals initiate the long-term redox alterations linked to CKMS—are they systemic or organ-restricted? Are these oxidative changes reversible, and do they differ between organ systems? Importantly, can a single, early-life antioxidant intervention mitigate or prevent the programming of CKMS in offspring?

In addition to oxidative stress, other central mechanisms implicated in CKMS programming—such as aberrant RAS activation [99,100], epigenetic dysregulation [101], gut microbiota imbalance [102], and altered nutrient-sensing pathways [16]—remain insufficiently explored, particularly regarding their interactions with oxidative stress [88].

Progress in this field is further hindered by heterogeneity in animal models, dietary compositions, timing of exposures, and the developmental windows studied, which limit the generalizability of findings. To bridge these gaps, future research should prioritize comprehensive profiling of oxidative stress responses, adopt multi-organ and systems-level investigative approaches, promote greater integration of DOHaD frameworks [193], and refine animal models to enhance translational applicability [26,88]. Longitudinal human studies are urgently needed to validate preclinical data and to identify critical windows for targeted intervention.

Ultimately, advancing our understanding of how maternal HFD exposure and oxidative stress contribute to the developmental programming of CKMS will be essential for shaping targeted dietary recommendations and public health strategies. Such efforts aim to promote healthier pregnancies and reduce the intergenerational transmission of CKMS risk.

## Figures and Tables

**Table 1:** Comparison of Oxidative Stress in CKM Programming vs. Overt CKMS.

Aspect	CKM Programming (Developmental Stage)	Overt CKMS (Clinical Disease Stage)
Timing	Fetal and early postnatal life	Postnatal through adulthood
Role	Developmental modulator	Pathogenic driver
Nature of Impact	Indirect, programming effects	Direct, damaging effects
Mechanisms Involved	Modulation of organogenesis Epigenetic programming Disruption of metabolic and immune set points	ROS/RNS-induced cellular injury Organ dysfunction Fibrosis and inflammation
Key Pathways	Aberrant RAS activation Epigenetic dysregulation Gut microbiota dysbiosis Dysregulated nutrient-sensing signals	Endothelial dysfunction Mitochondrial damage Lipid peroxidation NF-κB activation
Target Organs	Kidneys, heart, liver, adipose, gut (offspring)	Kidneys, heart, adipose, vasculature, pancreas, liver
Biomarkers	MDA, TBARS, F_2_-isoprostanes, 4-HNE, 8-OHdG (in offspring tissues) Altered transcriptome due to epigenetic shifts	Elevated systemic and tissue ROS Oxidative damage markers in plasma, urine, tissues
Downstream Effects	Predisposition to obesity, hypertension, CKD, type 2 diabetes Long-term structural and functional alterations without immediate pathology	Manifest disease: obesity, CVD, CKD, type 2 diabetes Organ injury and impairment

**Table 2:** Animal models of maternal high-fat-diet-induced CKMS programming related to oxidative stress.

Fat Fraction and Component	Pregnancy/Lactation	Species/ Gender	Age at Measure (Weeks)	Oxidative Stress	CKMS Phenotypes	Ref.
23% (Saturated fats)	Yes/Yes	SD rat/M	9	Increased renal iNOS expression	Kidney disease	[150]
24% (20% lard and 4% corn oil)	No/Yes	Wistar rat/F	24	Increased MDA, decreased nitrite levels, and decreased SOD, catalase, and GPx activities in the mesentery and plasma	Obesity, dyslipidemia, insulin resistance, and hypertension	[151]
24% (Lard)	No/Yes	Wistar rat/M	24	Increased MDA, decreased nitrite levels, and decreased SOD, catalase, and GPx activities in the mesentery and plasma	Obesity, dyslipidemia, insulin resistance, and hypertension	[152]
29% (Lard)	Yes/Yes	Wistar rat/M + F	24	Decreased activity of the antioxidant enzymes CAT, GPx and SOD in the liver	Obesity and hepatic steatosis	[153]
31% (Palm oil)	Yes/Yes	Wistar rat/M + F	13	Increased lipid peroxidation, increased 4-HNE in the liver, and reduced SOD activity in the kidneys	Hypertension	[154]
31% (Lard)	Yes/Yes	Wistar rat/M	14	Increased renal oxidative stress	Kidney disease and hypertension	[155]
33% (Lard)	Yes/Yes	C57BL/6 mice/M + F	20	Increased gp91^phox^, NOX4, and 8-OHdG expression in the pancreatic islets in M	Glucose intolerance, insulin resistance, and hepatic steatosis in M	[156]
35.5% (Lard)	Yes/Yes	CD-1 mice and GLUT4 heterozygous mice/M	24	Decreased Plin5 expression	Hypertension	[157]
43% (Saturated fats)	Yes/Yes	C57BL/6 mice/M	9	Increased renal 8-OHdG expression, decreased renal MnSOD expression	Kidney disease and increased renal triglyceride levels	[158]
43% (Saturated fats)	Yes/Yes	C57BL/6 mice/M	32	Increased renal NOX2 expression, nitrytyrosine, and 8-OHdG expression	Obesity, hyperglycemia, insulin resistance, and kidney disease	[159]
43% (Saturated fats)	Yes/Yes	C57BL/6 mice/M	32	Increased renal iNOS and 8-OHdG expression	Obesity, hyperglycemia, insulin resistance, dyslipidemia, and kidney disease	[160]
45% (Lard)	Yes/Yes	C57BL/6 mice/M	30	Decreased NO production and increased dihydroethidium staining in femoral artery, increased superoxide production in the liver	Obesity, hypertension, and endothelial dysfunction	[161]
45% (Lard)	Yes/Yes	SD rat/M	12	Increased TBARS levels, decreased GPx and SOD expression in the liver	Dyslipidemia and hepatic steatosis	[162]
45% (Saturated fats)	Yes/Yes	C57BL/6 mice/M	15	Increased ROS production and decreased glutathione levels in the liver	Dyslipidemia and hepatic steatosis	[163]
58% (Coconut oil)	Yes/Yes	SD rat/M	16	Decreased urinary NO level, increased renal oxidative stress	Kidney disease and hypertension	[164]
58% (Coconut oil)	Yes/Yes	SD rat/M	16	Elevated ADMA and reduced NO bioavailability	Hypertension	[165]
58% (Coconut oil)	Yes/Yes	SD rat/M + F	26	Increased renal 8-OHdG expression	Kidney disease	[98]

Studies stratified by fat fraction and component in maternal diet. SD = Sprague Dawley; GLU4 = glucose transporter 4; M = male; F = female; NO = nitric oxide; ROS = reactive oxygen species; SOD = superoxide dismutase; CAT = catalase; GPx = glutathione peroxidase.

**Table 3:** Summary of antioxidants used as reprogramming interventions in animal models of maternal high-fat-diet-induced CKMS programming.

Antioxidant Interventions	Species/ Gender	Age at Measure (Weeks)	Prevented CKMS in Offspring	Ref.
Resveratrol (50 mg/L) administered in drinking water during pregnancy and lactation	Wistar/M + F	3	Obesity, hyperlipidemia, hypertension	[181]
Resveratrol-supplemented diet (0.2% *w*/*w*) during pregnancy and lactation	C57BL/6J mice/M	14	Obesity, hyperlipidemia	[182]
Resveratrol (50 mg/L) administered in drinking water during pregnancy and lactation	SD rat/M	16	Kidney disease and hypertension	[164]
Resveratrol (50 mg/L) administered in drinking water during pregnancy and lactation	SD rat/M	16	Obesity	[171]
Quercetin (50 mg/kg/day) administered during pregnancy	C57BL/6J mice/M	24	Hypertension	[183]
Melatonin (5 mg/kg/day i.p.) administered during gestation and lactation	Wistar/M + F	3	Liver steatosis	[186]
Melatonin (0.01%) administered in drinking water during pregnancy and lactation	SD rat/M	16	Hypertension	[187]
N-acetylcysteine (300 mg/kg/day) administered during lactation	ICR-CD1 mice/M + F	13	Obesity and liver steatosis	[192]

Studies stratified by antioxidant interventions. SD = Sprague Dawley; M = male; F = female.

## Data Availability

Data are contained within the article.
